# Supplementary material for: Separability of Human Motor Memories during reaching adaptation with force cues
Source: PLoS Comput Biol. 2022 Oct 28;18(10):e1009966. doi: 10.1371/journal.pcbi.1009966 (PMC9645664; doi:10.1371/journal.pcbi.1009966)
Supplement: Attachment — Submitted filename: response.docx [file pcbi.1009966.s001.docx]

We want to thank the Editors for receiving our appeal favorably. Following the opportunity to re-submit the manuscript, we made the following change in the abstract:

*Judging by the breadth of our motor repertoire during daily activities, it is clear that learning different tasks is a hallmark of the human motor system. However, for reaching adaptation to different force fields, the conditions under which this is possible in laboratory settings have remained a challenging question. Previous work has shown that independent movement representations or goals enabled dual adaptation. Considering the importance of force feedback during limb control, here we hypothesised that independent cues delivered by means of background loads could support simultaneous adaptation to various velocity-dependent force fields, for identical kinematic plan and movement goal. We demonstrate in a series of experiments that indeed healthy adults can adapt to opposite force fields, independently of the direction of the background force cue. However, when the cue and force field were in the same direction but differed by heir magnitude, the formation of different motor representations was still observed but the associated mechanism was subject to interference. Finally, we highlight that this paradigm allows dissociating trial-by-trial adaptation from online feedback adaptation, as these two mechanisms are associated with different time scales that can be identified reliably and reproduced in a computational model.*

Besides, we added the Author Summary (which was previously labelled “Significant Statement”), and added after the reference list a short legend describing the supporting information.
