# Supplementary material for: Separability of Human Motor Memories during reaching adaptation with force cues
Source: PLoS Comput Biol. 2022 Oct 28;18(10):e1009966. doi: 10.1371/journal.pcbi.1009966 (PMC9645664; doi:10.1371/journal.pcbi.1009966)
Supplement: Attachment — Submitted filename: response_final.docx [file pcbi.1009966.s002.docx]

We wish to thank the Reviewers and the Editor for their careful examination of our work and highly valuable comments that allowed to improve the paper significantly. We were delighted that they received our work favourably and we are convinced that the revised version has improved. Please find below a point-by-point response to the different points that were raised along with references to the modifications that were made in the text.

Reviewer #1: This work investigates whether different background forces can, as cues, allow for separate, simultaneous adaptation to different force-fields applied during movement. In a set of experiments, this work finds evidence that first, oppositely-directed background forces can indeed cue opposing force-fields and, to a lesser extent, samely-directed background forces of different magnitudes can cue samely-directed force-fields of different magnitude. The authors use these data to demonstrate a model of adaptation with two timescales: one timescale representing online adaptation (within each movement) and another representing feedforward adaptation (from one trial to the next). The use of background forces as cues for different motor memories is a very interesting topic, the experiments are well-designed, the paper clear, the figures crisp. I have a number of questions regarding the framing/ interpretation of the results, and how the computational model relates to the experiment at hand.

Framing: As the manuscript discusses, previous work has shown that different force-fields can be cued by different prior or follow-up movements (e.g. refs 16,17). Here, the background forces are imposed before and remain on after the movement is executed. Could the present findings be understood within the concept of this previous work (or, maybe better, expanding the concept)? The common thread being, there is a motor plan before and after the movement that can act as a cue; whether this plan is to move from/to a different direction or to hold still against a different background load?

We agree that there is uncertainty relative to how the background force allows separating the motor plan. It can be the cue prior to movement, or knowledge that they have to hold their hand still against the load after movement completion, which in this case would be similar to the effect of a follow through action. Our data does not allow making this distinction. We highlight in the revised paper that it is an interesting question for prospective work:

*Page 17: “It is also possible that the difference across conditions was not confined to pre-movement phase, as participants knew that they had to control their movement and hold their hand still after movement end against a constant load, which may be used as a follow through action enabling separation of motor plans.”*

Lines 125-129 (and Figure 1d): because absolute angles are analyzed here, a smaller absolute angle could also be due to increased co-contraction (because of the added background load). How can we distinguish effects of actually learning the corresponding opposing fields vs. having smaller errors because of increased stiffness? (for this reason, I find the catch-trial findings eliciting oppositely-directed errors more convincing).

We completely agree that the reduction in initial angles from force field trials (Fig. 1d) are only indicative of adaptation insofar as an opposite effect is observed during catch trials (Fig. 1e). This was further stressed in the revised version of the paper:

Page 7: *“To establish that the compensation for the force field resulted from an adaptation of the controller instead of a default compensation strategy, […]”*

In similar lines: how can we distinguish between the part of the learning curves is due to stiffening vs. learning each force-field? In particular, the rapid component of the learning curve for path length (Figure 6c) could represent stiffening (especially later in movement) upon the introduction of the force-field. I suggest a brief discussion of these issues.

We agree that a potential role of co-contraction cannot be rejected firmly although we believe that our data do not support a strong case for this hypothesis. On the one hand the adaptation in Experiment 1 was mainly supported by the catch trials as highlighted in the comment above, while on the other hand we believe that a default use of co-contraction should be visible in both early and late movement parameters, thereby impacting initial angles and path length in a similar way. However, we acknowledge in the revised version that there remains uncertainty on this point:

Page 17: *“For instance, we cannot reject the possibility that participants used co-contraction in some phase of the experiment. We believe however that the impact of such strategy should be visible in the initial angle as it should be active at movement onset, which was not observed. In any case, a fuller quantification of the impact of feedback adaptation must still be established.”*

Figure 1 can benefit from the addition of a schematic illustrating the different phases of the experiment. While details can be inferred by a careful reading of the methods, a schematic could make this much easier.

Following the Reviewer’s suggestion, we added a schematic of the Experiment 1 in Panel a. The revised version of the legend is as follows:

Legend Fig. 1: *“illustration of the time course of the experiment: participants performed 3 blocks in one context (C1) including Left and Right background forces with catch trials (L, R, C, respectively), followed by a washout block (30 trials) and another series of 3 blocks in the opposite context (-C1).”*

Was the washout block (described in lines 428-431) analyzed? It would be particularly interesting to see if the different background forces during that period elicit performance reflecting the learning of one force-field or the other.

There was a tendency for a decay of the initial angles in absolute value towards 0 during the washout block, but it was not complete at the end of the 30 trials, so de-adaptation was not complete and participants kept anticipating partially the force field associated with the background load. However, a carry-over of the first context should bias the initial angles in the wrong direction during the second context, so this aspect should not impact our conclusions.

We added this information to the manuscript (Page 7): “*We also observed that the initial angles at the end of the washout block did not completely return to values corresponding to baseline, although there was a clear tendency for a decay in absolute value. Observe that it does not impact our results because upon switching context, the carry-over from the first context was in fact biasing the initial angles in the wrong direction.”*

Testing both conditions in all individuals is very reasonable. Were there any order effects however?

There was indeed an order effect that shifted all initial angles from catch trials up or down dependent on the first context experienced, but the difference reported in Fig 1f across contexts was highly consistent independent on the initial bias. We believe the Reviewer may have missed this information which was documented in the Results section:

Page 7: “*There was a directional bias corresponding to the order in which participants experienced the positive and negative contexts, such that those who performed trials in the positive context first tended to display larger values of initial angles, however the comparisons of initial angles during catch trials performed on each subgroup revealed similar differences. For the leftward background force, the differences between initial angles during catch trials across contexts were 9.8±1.8deg (positive first) and 15.5±2.3deg (negative first, mean±SEM). For the rightward background forces, these differences were -10.5±2.5 (negative first), and -11.7±2 (positive first).*”

Modeling: I don’t totally get how the two parts of this paper – different force-fields cued by different background loads on one hand, vs. a (very interesting) model encompassing both an online and offline component – blend together. The paper mentions that the data in Experiment 1 give a “novel opportunity to illustrate the model”. Can the authors elaborate on this? The model has been shown and tested in dedicated experiments in the authors’ recent work (ref. 33) – what regarding the present experiment design allows us to understand the model better? Is there something regarding background force cues that makes this dataset better suited to demonstrate the model than a garden-variety force-field adaptation task? From the other side, are there specific insights about learning multiple force-fields at the same time using force cues that are demonstrated through the model?

Following the Reviewer’s comment, we added motivation to the model. In our experience, the online adaptation mechanism still needs support, so any opportunity to highlight its impact is very important. In this case, the specificity of the paradigm allows highlighting it because trial-by-trial learning is slowed down by the cued, dual-adaptation task. In standard settings, trial-by-trial learning is difficult to dissociate from online adaptation because they are relatively fast and act in concert. In contrast, online adaptation is clearly visible in random adaptation tasks, where trial-by-trial adaptation is absent or limited.

Where the current paradigm offers a new view is that the random nature of the task promotes rapid and online adaptation while the cue still enables slower trial-by-trial adaptation, thereby separating the time scale of the two components and making them visible. This argument was added to the revised version of the manuscript:

Page 11: *“The data of Experiment 1 offered a novel opportunity to illustrate this model, because online and offline mechanisms may exhibit different timescales revealed by the dual adaptation task. In a standard adaptation scenario, short term effects of both trial-by-trial adaptation and online adaptation are confounded, whereas in fully random adaptation tasks, only the online component can be extracted. Here, the dual adaptation scenario slows down the trial-by-trial component, while the random alternation of force fields likely recruited the online component at a faster timescale, thereby dissociating their effects and making them visible.”*

Minor comments/questions

Abstract, Lines 25-28. This makes it sound like there was no interference in the opposite-direction case – however, to make that claim one needs to compare how quickly the two opposite force-fields are learned separately – instead, it might be better to say “increased interference”.

We agree and followed the Reviewer’s suggestion.

Not an important issue, but I was thinking the word “offline” to represent trial-to-trial adaptation might be confusing to some readers, as it may cue them to think of offline gains in learning/consolidation.

We opted to add the following line to clarify our definition and avoid confusion (page 11): *“By convention we refer to trial-by-trial updates in the controller as the “offline” adaptation mechanism.”*

The findings in Experiment 2 are definitely interesting – my first thought was that participants learn the average force-field, but the discussion addressed this well. Could participants be learning one field but scale their response by the force cue? Figure 2c suggests that the adaptation to the stronger force-field is privileged over adaptation to the smaller one – smaller errors – why would that be?

We share the Reviewer’s curiosity for this phenomenon and are currently pursuing follow up experiments to elucidate what happens. To scale the force field with the magnitude of the cue was in our initial guess the obvious response (as for instance inertia scales with mass for constant gravity, we expected Exp. 2 would be straightforward). In contrast to Fig. 2c however, the adaptation to the heavy FF is impaired upon introduction of the light one, which suggests also an effect linked not to the heavier field in absolute but to the one that cause larger errors, which in Exp 3 was the light one.

Our preliminary observations indicate that learning opposite force fields with cues of different magnitude was impossible, whereas the contrary (different magnitudes with opposite cues) was difficult but possible at least for some participants. We hope to communicate these results to the community in a near future.

Lines 472-473: is it necessary to truncate traces at 600ms to calculate path length? What fraction of trials was completed by that time?

It is difficult to determine whether a trial is fully completed or not since there always remain a residual velocity and a criterion must be used. To give quantitative information instead we report the interquartile range of hand speed (including x and y components) at that time (Page 23): *“The interquartile range of absolute forward velocity at that time across all participants from Experiment 1 was [0.01, 0.044]m/s.”*

Line 586: which data were used for that purpose?

The grand average across all traces were used as these traces are used only for illustration purpose.

Reviewer #2: This study investigates whether distinct background forces are sufficient cues to allow for dual adaptation to opposing forces fields. The authors found partial dual adaptation, errors reduce across training and expected catch trials are produced, when the background forces are in opposing directions although this is less the case when the background forces are in the same direction but different magnitudes, likely due to interference. The papers also demonstrates that the time courses of dual adaptation to FF with opposing backgrounds appears to be made of a combination of offline and online adaptation. The results are very interesting and a nice contribution to our understanding of the contextual cues that brain needs to concurrently create distinct motor memories. I particularly like Experiment 3; which is a somewhat novel way (as far as I know) of exploring interference and dual adaptation.

I was informed that this is a revision, but I wasn’t one of the original reviewers and didn’t see the reviewers or replies. But I don’t have much to add but only a few comments.
The figures are great, and I appreciate the individual data for the catch-trials. But I think the paper would benefit from seeing individual data for training phases, especially for Experiment 2, where the effect could be driven by a subset of participants especially since the sample size is bordering on too small. I know individual curves may be too messy, so maybe fitting an exponent decay with an asymptote and plot the rate of change and asymptote values. Such fits are more sensitive for looking at differences in in learning rates (across groups, as a function of interference etc) than just averaging the first block of trials (which these authors don’t do here but is fairly common).

We wish to highlight that the results of Experiment 2 were associated with power > 0.8 and were furthermore reproduced in Experiment 3. Thus, although we like experiments with a lot of subjects, we feel that the results are not underpowered. To address the Reviewer’s comment specifically, we calculated individual slopes for the second phase of Experiment 2 for heavy and light trials, and found that the slopes were clearly more negative and significant for a majority of participants during light trials, whereas they tended to be distributed around zero for the heavy ones. These results further confirm the interaction result shown in the linear mixed model, and emphasize that it was not driven by a minority of participants. They were added to Figure 2 (panel d, legend) and associated text as follows:

Page 9*: “This tendency was captured for individual participants by fitting linear models to the same data (starting at trial 21, after the initial rapid phase). The individual slopes were clearly more negative and were significant in a majority of participants for light trials (Fig. 2d, L), while the slopes for heavy trials were distributed around zero and only a few were significant. Paired comparison of the slopes across heavy and light trials were significant (|t­_13_| = 3.5, P = 0.003).”*

I can imagine that how early the background force is experienced prior to movement onset could make a difference to how useful the cue is, especially when differentiating between a small and large background forces, although this is not mentioned. In fact, I had no idea when or what event triggered the background force until I read the methods at the end and I’m still not 100% sure. The methods indicate the background force was applied (across 500 ms ramp) prior to target onset but before participants stabilized their hand onto the home to start the movements. What does stabilisation entail? My understanding is that people acquire the start target, and that then the background force ramps up but is there an additional requirement that they had to keep their hand within a window of the start position (make sense) which would have been a “challenge” since they have to overcome the background force. How long did it take for participants to stabilize (given that the background force should have made it more challenging)? And likewise, what was the average amount of time people experience the full force of the background prior to movement onset. Once the background force ramped up, how long until the target appears. Did the amount of time that background force was experience differ for the different magnitudes of forces given that it would been easier to stabilise the hand at the start position for the light background force than the heavy one.
It may be useful to describe background force timing a bit earlier than the methods or make use of Fig 1 and its legend to provide a bit information about this timing relative to target onset and start stabilization. The timecourse for b and c looks more like a schematic, so not sure whether to rely on it to indicate that actual time that the full background force was applied prior to movement onset (0).

The procedure was clarified: they had to stabilize against the background load which was applied prior to their initial movement to the start target:

Page 20: “*This background load was applied independently of the cursor location, thus participants first had to acquire the start target with the background load on, or return to it, against the load. The random delay comprised between 2 and 4 seconds was counted only if the cursor was stable in the start target, meaning that participants awaited the go cue while constantly compensating for the background load.”*

Line 385, the term “interrogations” seems odd. I understand what you mean but its not match the context and has a negative connotation.

The sentence was modified, *interrogations* was replaced by *open* *questions*

Line 26. The “t” in their is missing

Corrected.
